# Energy from Negentropy of Non-Cahotic Systems

**DOI:** 10.3390/e20020113

**Published:** 2018-02-09

**Authors:** Piero Quarati, Antonio M. Scarfone, Giorgio Kaniadakis

**Affiliations:** 1 Dipartimento di Scienza Applicata e Tecnologia (DISAT-SCUDO)-Politecnico di Torino, 10129 Torino, Italy; 2 Istituto Sistemi Complessi (ISC–CNR) c/o Politecnico di Torino, 10129 Torino, Italy; 3 Istituto Nazionale di Fisica Nucleare (INFN)-Sezione di Torino, 10125 Torino, Italy

**Keywords:** negentropy, non-ideal systems, many-body correlations

## Abstract

Negative contribution of entropy (negentropy) of a non-cahotic system, representing the potential of work, is a source of energy that can be transferred to an internal or inserted subsystem. In this case, the system loses order and its entropy increases. The subsystem increases its energy and can perform processes that otherwise would not happen, like, for instance, the nuclear fusion of inserted deuterons in liquid metal matrix, among many others. The role of positive and negative contributions of free energy and entropy are explored with their constraints. The energy available to an inserted subsystem during a transition from a non-equilibrium to the equilibrium chaotic state, when particle interaction (element of the system) is switched off, is evaluated. A few examples are given concerning some non-ideal systems and a possible application to the nuclear reaction screening problem is mentioned.

## 1. Introduction

In thermodynamics and in statistical mechanics, the entropy enters, with a central role, into all laws concerning systems in states of equilibrium and non-equilibrium: in reversible and irreversible transformations and phase transitions, in Boltzmann and Gibbs classical and quantum extensive and non-extensive statistics.

Entropy can be expressed as a total, compact, positive expression always increasing in isolated systems during its time evolution towards equilibrium. Often, entropy is composed by a few different terms and, when the system is not in an equilibrium chaotic state, at least one of them represents a negative contribution to its evaluation.

The negative entropy (negentropy) contribution is present because of many different reasons like the quantum Pauli exclusion or boson inclusion principles, the many-body correlations and interactions among the single elements composing the system. Quantum exclusion-inclusion effects as well as correlations and/or interactions produce order to the system decreasing the value of the total entropy [1].

It is interesting to study the negative entropy contribution, different from the positive one, because negentropy represents a stored mobilizable energy in organized systems. This quantity can be spontaneously transferred or exchanged among the elements of the system, or from these elements to an inserted subsystem, or from an organized environment to elements of a system [2,3]. In a non-ideal system, as for instance gas, plasma or liquid and solid metal, composed by interacting or correlated elements in a stationary non-equilibrium (long-life metastable) state, some energy can be transferred to other particles of a subsystem (as for instance deuterons or very light nuclei) implanted into its volume size. The relative energy of the reacting deuteron nuclei can be increased by negentropy of a given number of the system elements, allowing fusion reactions against Coulomb repulsion more easily [4].

Experimental measurements of nuclear fusion rates of deuterons inserted in a liquid or solid metal matrix, impinged by accelerated deuterons of a given kinetic energy much greater than expected, can be explained by means of the role of negentropy of the matrix. Other examples and applications can be found in astrophysical plasmas, where often measured reaction rates are greater than expected [5,6].

The creation of order and energy of high value requires non-equilibrium conditions. If negative entropy of the single elements is responsible for the energy transfer, we expect that small islands of disorder, where negative entropy is spent, and islands of order, where negative entropy is received, will be created. Without any action from outside the system, switching off the interaction among the elements, makes the entropy increase or negative entropy decrease.

Our work is close to the papers by Sato [7,8] and by Yi-Fang Chang [9,10], but differs because we evaluate the energy that can be transferred by means of negentropy to induce and facilitate processes that without this transferred energy would be absent. However, our method is not an alternative to that of Sato and that of Yi-Fang Chang but rather allows the study of particular situations in which the previous methods are less appropriate. Sato has generalized the definition of negentropy that becomes valid under general situations: negentropy represents the potential of work defined by Kullback-Leibler information [11,12]; this is more appropriate than being defined by the difference of entropies.

In this work we calculate the amount of energy that a single element of a system can transfer to other internal elements or clusters of elements spending entropy-lowering (by means of negentropy) when the interaction or the correlations among the elements are switched off or when their intensity is lowered. A few evaluations are reported concerning non-ideal molecular gas, warm dense matter (pseudo white dwarf) and nuclear matter while possible application to the screening of nuclear reactions in plasmas is indicated.

We show the constraints to which negative and positive contributions of entropy and free energy are submitted, explain how negentropy is linked to correlations among the elements of a system and report how energy fluctuation is due to negentropy and therefore to correlations.

In Section 2 we define the different positive and negative contribution of entropy. In Section 3 we report the suggestion by Clausius and Helmholtz concerning the splitting of entropy and free energy in free and bound contribution. In Section 4 we present a different splitting of entropy and free energy while in Section 5 we discuss the corresponding energy transfer mechanism. In Section 6 we give an explicit example to evaluate the energy that can be exported by some elements of the system to an inserted element of a subsystem in a non-ideal system; in Section 7 we report a few applications with numerical examples. Conclusions are given in Section 8.

## 2. The Different Entropy Contributions

Let us consider a statistical system of elements (for instance laboratory plasma, astrophysical or stellar plasma, solid and liquid metal, non-ideal gas, warm dense matter; the system can also be an environment or a medium) that contains a subsystem (implanted nuclei, accelerated ions through the system, special cluster of different particles). The system is composed of *N* elements much larger than the number of elements of the subsystem. The subsystem interacts with a finite number *M* of elements of the system with M≪N. For instance, in a time Δt during successive elastic collisions or after a mean free path, the subsystem enters into an interaction with *M* elements of the system. We assume that the system is not in a global thermodynamic equilibrium; it is not in a full disorder chaotic state but has a certain degree of order. To be more precise, the system is in a stationary state with a long lifetime (metastable state), its entropy is the sum of a positive and a negative contribution because correlations and/or interactions are present, which are responsible, for isolated systems, for a lower value of entropy. The negative terms are expression of the order and represent the correlation entropy contribution. Without any action from outside of the system, by switching off the interaction, we impose the entropy to increase or the negative entropy to reduce.

Of course, the evolution in time of entropy is always positive for isolated systems; this is the same for the entropy of an ensemble in equilibrium. For instance, a classical ideal gas is always the sum of positive contributions.

However, if we introduce particle correlations, for instance and for simplicity, for quantum effects like the Pauli exclusion or the inclusion-exclusion principle, that give a sort of order to the gas, the related contributions to entropy are negative and the entropy decreases compared to its value for classical, uncorrelated ideal gas.

Therefore, a system with correlations or interactions among the *N* elements, with a given potential energy, has negative contribution in the entropy expression:

(1) $$\begin{array}{c} S=S^{\mathrm{ideal}}-S^{\mathrm{corr}}\;, \end{array}$$

where $S^{\mathrm{ideal}}$ is the maximum value of entropy that is the entropy of the equilibrium state and $S^{\mathrm{corr}}$ is the contribute due to the correlations, $S^{\mathrm{corr}}$ was also called by Gibbs [13] “capacity of entropy or negentropy”, by Brillouin [14] “input or information”, by Obukhov [15] “deficiency of entropy” and by Sato [7,8] “potential of work or negentropy”.

If the interaction is a weak short-range, two-body interaction, the equilibrium distribution function is still Maxwellian and the entropy is the sum of positive terms only.

A state of thermal equilibrium is chaotic at zero value of controlling parameters (an example of a controlling parameter of interest here is the interaction two-body potential U(r) as discussed in Section 7) and entropy is a measure of chaoticity or deviation from equilibrium of a state.

Non-equilibrium state does not remain constant after isolation because of irreversible processes that are responsible for transitions to a state of equilibrium on the same energy shell. A stationary state of non-equilibrium of a system is a time invariant state of an open system. The entropy of an equilibrium state is greater than in state of non-equilibrium with non-zero value of control parameters. Moreover, we can compare states of a different order (or chaoticity) only if they belong to the same energy shell.

## 3. Clausius and Helmholtz Suggestion

Following Clausius and Helmholtz, it seems useful to divide the energy of a system in two parts $E^{b}$ and $E^{f}$, where *b* is for bound and *f* for free [16,17,18]:

(2) $$\begin{array}{c} E=T\;S+F=E^{b}+E^{f}\;, \end{array}$$

with $E^{b}=T\;S$ the part of energy bound in thermal motion of molecules and $E^{f}=F$ the part of energy free to perform work and coincides with the thermodynamical free energy.

In a system at equilibrium, with a given volume *V*, temperature *T* and number of elements *N*, the value of entropy *S* is maximum and the value of free energy *F* is minimum. The more work that can be produced, the greater the value of the entropy.

Like energy, entropy can also be divided into a bound and a free part

(3) $$\begin{array}{c} S=S^{b}+S^{f}\;, \end{array}$$

where $S^{b}$ is the entropy bound in the microscopic motion of molecules or the *N* elements of the system, while $S^{f}$ is the entropy available for information processing or to produce work outside the system. We argue that $S^{f}$ can also be exchanged among the elements or group of elements of the system or with elements of an inserted subsystem.

Because of the splitting of *S*, it would be more correct, in place of the above definitions $E^{b}=T\;S$ and $E^{f}=F$, to write:

(4) $$\begin{array}{c} {\tilde{E}}^{b}=T\;S^{b}\;\mathrm{and}\;{\tilde{E}}^{f}=T\;S^{f}+F\;. \end{array}$$

Then, the energy available to produce work becomes ${\tilde{E}}^{f}$. In addition and for completeness, it would be better also to split the free energy *F* in:

(5) $$\begin{array}{c} F=F^{b}+F^{f}\;, \end{array}$$

where the quantities $F^{b}$ and $F^{f}$ can be understood as it follows: at equilibrium *F* is minimum ($F^{eq}=F^{b}$ which is a negative quantity) and $F^{f}=0$. Moving toward non-equilibrium state, taking *T*, *V* and *N* constants, the positive quantity $F^{f}$ increases and the total *F* increases too.

By grouping all the terms with index *b* together and all the terms with index *f* together we obtain:

(6) $$\begin{array}{c} {\bar{E}}^{b}=T\;S^{b}+F^{b}\;,\;\mathrm{and}\;{\bar{E}}^{f}=T\;S^{f}+F^{f}\;, \end{array}$$

with

(7) $$\begin{array}{c} E=E^{b}+E^{f}={\tilde{E}}^{b}+{\tilde{E}}^{f}={\bar{E}}^{b}+{\bar{E}}^{f}\;. \end{array}$$

Therefore, the energy bound to microscopic motion is ${\bar{E}}^{b}$ and the energy available for work is ${\bar{E}}^{f}$. Clearly, at equilibrium total energy is ${\bar{E}}^{b}$ and ${\bar{E}}^{f}=0$.

If during the transition from equilibrium to non-equilibrium, or vice versa, temperature, volume and number of elements do not change, then $S^{b}$ and $F^{b}$ do not change, as well as ${\bar{E}}^{b}$.

## 4. A Different Subdivision of Free Energy and Entropy

In the following, we consider a system of independent elements and a system with elements correlated or interacting through a potential and therefore with an entropy value lower than that of a system with independent elements. We focus our attention on the subdivision of entropy and free energy into the contributions related to equilibrium, where contributions to produce work are absent if *V*, *N* and *T* remain constant, and into the contributions due to the presence of correlations or interactions among the elements of the system. These are the contributions that can produce work and can be spent internally and locally between elements of the system or can be spent outside the system or to an inserted subsystem. In this way, the system will lose order and become more chaotic.

We may divide the total entropy in two parts: one positive and one negative. Accordingly and differently from Decomposition (3), we consider:

(8) $$\begin{array}{c} S=S^{+}-S^{-}\;, \end{array}$$

with $S^{-}>0$.

The contribution $S^{-}$ is due to correlations or interactions and brings order to the system; it can also be thought of as an environment whose entropy at global thermodynamic equilibrium is given by $S^{+}$. Negentropy $S^{-}$ is available to transfer energy to other subjects and to do work.

Let us divide also the free energy *F* into two parts:

(9) $$\begin{array}{c} F=F^{+}-F^{-}\;, \end{array}$$

with $F^{-}>0$; the part $F^{+}$ can do work.

Since, the energy of the system is E=F+TS, we may write:

(10) $$\begin{array}{c} E=\left(F^{+}-F^{-}\right)+T\;\left(S^{+}-S^{-}\right)\;. \end{array}$$

As a consequence, not all the free energy *F* contributes to make work and the entire quantity of T,S does not represent work.

Comparing Equations (8) and (10) with Equations (3) and (5), we can write the following relations

(11) $$\begin{array}{c} S^{b}=S^{+}\;,\;\mathrm{and}\;S^{f}=-S^{-}\;, \end{array}$$

as well as

(12) $$\begin{array}{c} F^{b}=-F^{-}\;,\;\mathrm{and}\;F^{f}=F^{+}\;, \end{array}$$

so that

(13) $$\begin{array}{c} {\bar{E}}^{b}=T\;S^{+}-F^{-}\;,\;\mathrm{instead}\;\mathrm{of}\;E^{b}=T\;S\;, \end{array}$$

and

(14) $$\begin{array}{c} {\bar{E}}^{f}=-T\;S^{-}+F^{+}\;,\;\mathrm{instead}\;\mathrm{of}\;E^{f}=F\;. \end{array}$$

## 5. Transfer of Energy

A system in global thermodynamic equilibrium with entropy $S=S^{+}$ does not have energy to spend outside. On the other hand, a system, with entropy $S=S^{+}-S^{-}$ can transfer energy to an inserted subsystem that, entering in interaction during a mean free path with *M* elements, can receive an average energy from negentropy up to the quantity $M\;T\;S^{-}/N$. The system loses order, and the subsystem gains energy.

If the *M* elements of the system that interact with the subsystem transfer their available energy to the subsystem at constant temperature *T*, the variation of energy is:

(15) $$\begin{array}{c} \Delta E=T\;\Delta S+\Delta F\;. \end{array}$$

For simplicity of notation we define entropy S, energy E and free energy F per element, that is S=S/N, E=E/N and F=F/N.

The total energy variation of the system, after transfer of some amount of energy to the subsystem with a transition from the initial state 1 to the final state 2, is:

(16) $$\begin{array}{cc} \Delta E=E_{2}-E_{1} & =T\;\left(S_{2}^{+}-S_{1}^{+}\right)-T\;\left(S_{2}^{-}-S_{1}^{-}\right)+\left(F_{2}^{+}-F_{1}^{+}\right)-\left(F_{2}^{-}-F_{1}^{-}\right)\\ & =T\;\left(\Delta S^{+}-\Delta S^{-}\right)+\Delta F^{+}-\Delta F^{-}\\ & =\left(-T\;\Delta S^{-}+\Delta F^{+}\right)+\left(T\;\Delta S^{+}-\Delta F^{-}\right)\\ & =\Delta E^{f}+\Delta E^{b}\;. \end{array}$$

Toward the equilibrium, the free energy *F* must diminish and the entropy *S* must increase, therefore ΔF<0, ΔS>0, that is $\Delta F^{+}<\Delta F^{-}$ and $\Delta S^{+}>\Delta S^{-}$, or:

(17) $$\begin{array}{ccc} F_{2}^{+}-F_{2}^{-}- & < & F_{1}^{+}-F_{1}^{-}\;, \end{array}$$

(18) $$\begin{array}{ccc} S_{2}^{+}-S_{2}^{-} & > & S_{1}^{+}-S_{1}^{-}\;. \end{array}$$

If the system loses energy (ΔE<0, i.e., $\Delta E^{b}+\Delta E^{f}<0$), the work to the subsystem is made by $F^{+}$ and/or $S^{-}$ and

(19) $$\begin{array}{c} \Delta F^{+}-\Delta F^{-}<T\;\left(\Delta S^{-}-\Delta S^{+}\right)\;. \end{array}$$

The total exchanged energy $E_{\mathrm{ex}}$ between the system and the subsystem, at constant temperature, is:

(20) $$\begin{array}{c} E_{\mathrm{ex}}=M\;\left(-T\;S_{1}^{-}+F_{1}^{+}\right)\;, \end{array}$$

so that the residual total energy $E_{\mathrm{res}}$ of the system becomes:

(21) $$\begin{array}{ccc} E_{\mathrm{res}} & = & E_{1}-E_{\mathrm{ex}}\\ & = & N\;\left[T\;\left(S_{1}^{+}-S_{1}^{-}\right)+F_{1}^{+}-F_{1}^{-}\right]-M\;\left(-T\;S_{1}^{-}+F_{1}^{+}\right)\\ & = & -\left(N-M\right)\;T\;S_{1}^{-}+\left(N-M\right)\;F_{1}^{+}+N\;\left({T\;S_{1}^{+}-F}_{1}^{-}\right)\;. \end{array}$$

In the transfer of energy from the initial state to the final state, all the above relations should be satisfied because of the increase of total entropy *S* and the decrease of *F*.

The important point is that the expression to evaluate the transferred energy on the work that can be performed is not $E=F=F^{+}-F^{-}$. It is instead given by the difference of the positive free energy $F^{+}$ and the negentropy contribute $T\;S^{-}$. This means that a part of entropy also contributes to perform work and a part of free energy contributes to produce heat or non-information. $F^{+}$ and $S^{-}$ are different from zero when the elements of the ensemble are submitted to correlations or interactions. These two quantities go to zero when the system goes to equilibrium. In this case, the remaining quantities different from zero are $F^{-}$ and $S^{+}\equiv S^{\mathrm{eq}}$, while $S^{-}$ can be identified with negentropy or lowering of entropy $S^{-}=S^{\mathrm{eq}}-\Delta S$.

In conclusion, let us describe two examples to evaluate the energy that can be exported or imported by the system during a process.

We recall that sometimes [9,10], the variation of entropy of a system is divided into internal $S^{\mathrm{int}}$ and external $S^{\mathrm{ext}}$ entropy (the internal entropy is related to irreversible processes):

(22) $$\begin{array}{c} \Delta S=\Delta S^{\mathrm{ext}}+\Delta S^{\mathrm{int}}\;, \end{array}$$

$\Delta S^{\mathrm{int}}\geq 0$ is required by the second law of thermodynamics.

- Case of exporting entropy

We study first the conditions required to prepare a system with an entropy lower than its greatest value when at equilibrium. Since the order increases if the entropy decreases, the system can have lower entropy not only exporting entropy but also importing negentropy. Of course, the system that exports negentropy increases its entropy.

We want ΔS<0 to increase the order of the system, so that:

(23) $$\begin{array}{c} -\Delta S^{\mathrm{ext}}>\Delta S^{\mathrm{int}}\geq 0\;. \end{array}$$

This expression represents the thermodynamic condition for self-organization. We must export more entropy than the entropy variation for the irreversible internal transformations. In fact, from relation:

(24) $$\begin{array}{c} \Delta S=\left(S_{2}^{\mathrm{ext}}-S_{1}^{\mathrm{ext}}\right)+\Delta S^{\mathrm{int}}<0\;, \end{array}$$

and using $\Delta S^{\mathrm{int}}\geq 0$, we get the condition $S_{1}^{\mathrm{ext}}>S_{2}^{\mathrm{ext}}$, that is, the external entropy should decrease.

We repeat the relations of the above transition by using our definition of entropy (8), that is:

(25) $$\begin{array}{c} \Delta S=\Delta S^{+}-\Delta S^{-}<0\;. \end{array}$$

By identifying

(26) $$\begin{array}{c} \Delta S^{+}\equiv \Delta S^{\mathrm{int}}\geq 0\;, \end{array}$$

and

(27) $$\begin{array}{c} \Delta S^{-}\equiv -\Delta S^{\mathrm{ext}}\;, \end{array}$$

the condition (25) gives

(28) $$\begin{array}{c} 0\leq \Delta S^{-}<\Delta S^{+}\;. \end{array}$$

Therefore, negentropy increases, the order increases and the positive contribution must increase in a quantity to keep valid the condition ΔS<0.

- Case of importing entropy

The opposite case is when the system increases its entropy ΔS>0. Since the order decreases if the entropy increases, the system can have higher entropy not only importing entropy but also exporting negentropy.

We want

(29) $$\begin{array}{c} \Delta S^{\mathrm{ext}}>-\Delta S^{\mathrm{int}}\;, \end{array}$$

that is, internal entropy increases, therefore external entropy may decrease quite a lot despite total entropy increasing. However, the disorder shall increase.

In this case, by using our definition, we have:

(30) $$\begin{array}{c} \Delta S^{+}>\Delta S^{-}\;, \end{array}$$

that is assured if $\Delta S^{-}<0$ since $\Delta S^{+}\equiv \Delta S^{\mathrm{int}}\geq 0$. Therefore, positive contribution increases more than negative contribution is exported. Entropy increases because negentropy is exported.

## 6. An Explicit Example to a Non-Ideal System

We define free energy *F* and entropy *S* for a non-ideal system of elements interacting by the two-body potential U(r) following [19]. The two quantities $F^{-}$ and $F^{+}$ reads:

(31) $$\begin{array}{c} F^{-}=k\;T\;\ln A\left(T\right)\;, \end{array}$$

(32) $$\begin{array}{c} F^{+}=k\;T\;N\;n\;B\left(T\right)\;, \end{array}$$

with n=N/V, where

(33) $$\begin{array}{c} A\left(T\right)=\frac{V^{N}}{N!}{\left(\frac{2\;\pi \;m\;k\;T}{h^{2}}\right)}^{3\;N/2}\;, \end{array}$$

(34) $$\begin{array}{c} B\left(T\right)=2\;\pi \int_{0}^{\infty }\left(1-\exp\left(-\frac{U(r)}{k\;T}\right)\right)\;r^{2}\;dr\;, \end{array}$$

and from the thermodynamical relation

(35) $$\begin{array}{c} S=-{\left(\frac{\partial F}{\partial T}\right)}_{V,N}\;, \end{array}$$

and using the Decompositions (8) and (9), we have

(36) $$\begin{array}{c} S^{+}={\left(\frac{\partial F^{-}}{\partial T}\right)}_{V,N}\;,\;\;S^{-}={\left(\frac{\partial F^{+}}{\partial T}\right)}_{V,N}\;. \end{array}$$

Therefore, we get

(37) $$\begin{array}{c} T\;S^{+}=k\;T\;\ln A\left(T\right)+\frac{3}{2}\;N\;k\;T\;, \end{array}$$

(38) $$\begin{array}{c} T\;S^{-}=k\;T\;N\;n\;B\left(T\right)-N\;n\;C\left(T\right)\;, \end{array}$$

where

(39) $$\begin{array}{c} C\left(T\right)=2\;\pi \int_{0}^{\infty }U\left(r\right)\;\exp\left(-\frac{U(r)}{k\;T}\right)\;r^{2}\;dr\;, \end{array}$$

so that, the energy becomes

(40) $$\begin{array}{c} E=F+T\;S=N\;\left(\frac{3}{2}\;k\;T+n\;C\left(T\right)\right)\;. \end{array}$$

We consider the two following cases:

1. Transition from nonequilibrium to equilibrium by switching off the interaction: from the state (T,V,N,U(r)≠0) to $\left(\hat{T},\;V,\;N,\;U\left(r\right)=0\right)$. The energy difference is
  (41) $$\begin{array}{c} \Delta E=N\;\left(\frac{3}{2}\;k\;\Delta T+n\;\Delta C\left(T\right)\right)\;, \end{array}$$
  with $\Delta T=\hat{T}-T$.
  The second term ΔC(T) derives from terms containing the interaction U(r).
  Let us consider a system in interaction whose potential can be modelled by an average energy value 〈U(r)〉 with an interaction radius equal to $R_{c}$. Then, Equations (37) and (38) still hold where now
  (42) $$\begin{array}{c} B\left(T\right)=\frac{2}{3}\;\pi \;R_{c}^{3}\;\left(1-\exp\left(-\frac{\langle U(r)\rangle }{k\;T}\right)\right)\;, \end{array}$$
  (43) $$\begin{array}{c} C\left(T\right)=\frac{2}{3}\;\pi \;\langle U\left(r\right)\rangle \;R_{c}^{3}\;\exp\left(-\frac{\langle U(r)\rangle }{k\;T}\right)\;. \end{array}$$
  When the interaction is switched off, the same amount of energy calculated above can transfer to one element of the system itself (or a cluster of elements) by at most M≪N elements during a time Δt between two successive elastic collisions while the element performs a mean path length. The accelerated element (or cluster) can later give back the acquired energy to other elements. The energy of an element, during the non-equilibrium to equilibrium transition with switching off the interaction or correlation U(r), fluctuates of the order of $T\;S^{-}/N$ and the energy per particle gained before is $\frac{3}{2}\;k\Delta T+n\;\Delta C\left(T\right)$. The bulk properties of the system do not change, however, locally, a modification of the features of the site can be observed.
2. Transition from equilibrium state (T,V,N,U(r)=0) to the equilibrium state $\left(T,\hat{V},\;N,\;U\left(r\right)=0\right)$:
  (44) $$\begin{array}{c} \Delta F\equiv \Delta F^{-}=N\;k\;T\;\ln\left(\frac{\hat{V}}{V}\right)=-p\;\Delta V\;, \end{array}$$
  where $\Delta V=\hat{V}-V$ and pΔV is the work performed outside the system. This relation implies $\Delta S=\Delta S^{+}$, so that
  (45) $$\begin{array}{c} Q=-N\;k\;T\;\ln\left(\frac{\tilde{V}}{V}\right)\;, \end{array}$$
  is the heat received from the outside to maintain *T* constant and ΔE=0.

## 7. Some Possible Applications

We report a few applications to several non-ideal systems described by simple models just to have an indication of the order of magnitude we might expect from the negentropy effect described in the previous sections.

- Molecular gas

We consider a non-ideal gas with two-body interaction modelled by $\langle U\left(r\right)\rangle =2\times 10^{-2}\;\mathrm{eV}$ with $R_{c}=5\times 10^{-8}\;\mathrm{cm}$. At temperature T=300K, corresponding to the thermal energy $k\;T=2.6\times 10^{-2}\;\mathrm{eV}$ per donor, and a particle density $n=10^{20}\;{\mathrm{cm}}^{-3}$, the negentropy transfer of energy is $T\;S^{-}=4\times 10^{-6}\;\mathrm{eV}$. Otherwise, changing the average potential value in $\langle U\left(r\right)\rangle =10^{-1}\;\mathrm{eV}$ and the particle density in $n=10^{22}\;{\mathrm{cm}}^{-3}$, we obtain $T\;S^{-}=5\times 10^{-2}\;\mathrm{eV}$.

- Warm dense matter (a pseudo white dwarf) and nuclear matter

We consider a non-ideal neutral gas, composed by a background of α-particles (He${}^{4}$ nuclei) and a degenerate Fermi gas of electrons. The system looks like a pseudo white dwarf made of a warm, dense matter. Let us focus our attention to the inert α-particle gas with the following parameters: $n=10^{30}\;{\mathrm{cm}}^{-3}$; $T=10^{7}\;K$, corresponding to the thermal energy kT=860eV, and $R_{c}=10^{-10}\;\mathrm{cm}$ (the range of average correlated α−α screened potential).

We evaluate the energy that a single element can transfer as a function of 〈U(r)〉/kT (the value of 〈U(r)〉 depends on the assumption of the screening model) given by (). If we take $\langle U\left(r\right)\rangle /k\;T=10^{-2}$, each element can transfer 18eV of energy that reduces to 9eV if $\langle U\left(r\right)\rangle /k\;T=5\times 10^{-3}$.

During a mean free path λ inside the system, an α-particle or an ion or deuteron inserted into the system interacts with M≪N elements and can transfer to it the energy given by

(46) $$\begin{array}{ccc} \frac{M}{N}\;T\;S^{-} & = & n\;\pi \;R_{c}^{2}\;\lambda \;T\;S^{-}\\ & = & \frac{2}{3}\;\pi^{2}\;\frac{R_{c}^{5}}{\sigma }\;n\;k\;T\;\left[1-\left(1+\frac{\langle U(r)\rangle }{k\;T}\right)\;\exp\left(-\frac{\langle U(r)\rangle }{k\;T}\right)\right]\;. \end{array}$$

We assume a mean free path $\lambda =10^{-9}\;\mathrm{cm}$ and recalling that σ=1/(nλ), the elastic cross section is $\sigma =10^{-21}\;{\mathrm{cm}}^{2}$. The value of *M* is about 30 particles so that the total energy transferable is 550eV if $\langle U\left(r\right)\rangle /k\;T=10^{-2}$ and reduces to 280eV if $\langle U\left(r\right)\rangle /k\;T=5\times 10^{-3}$.

This amount of energy does not change the bulk property of the system, nevertheless it can improve the probability of an α−α or an α-nucleus fusion reaction inside the pseudo white dwarf. A description and result analogous to those of a pseudo white dwarf can be made for a system of nuclear matter where the kinetic energy of an element (nucleon) could be increased by means of *M* elements donors of negentropy.

- Liquid metals

A great number of liquid metals have a negentropy of about 2.5eV [20,21]. An inserted subsystem can receive a few hundred eV when it interacts with 100 elements during a mean free path.

For instance, in liquid metals with implanted deuterons, the fusion reaction rate with deuterons accelerated through the metal increases because the term $M\;T\;S^{-}$ increases the kinetic energy of the colliding nuclei [4]. This permits an easier passage through the Coulomb barrier by the tunnel effect. This effect is in addition to electron screening, stopping power and other microscopic effects.

- Nuclear reaction in plasmas

Another important application concerns the screening of nuclear reactions in plasmas slightly far from equilibrium. It is well known that the rate of nuclear fusions in a plasma grows with the plasma screening effect. In the weak screening regime with plasma parameter Γ<1, Salpeter’s approach works. A colliding pair of equal nuclei with relative kinetic energy receive an extra kinetic energy given by ${\left(Z\;e\right)}^{2}/\lambda_{D}$, where the Debye-Hückel length is $\lambda_{D}^{2}=k\;T/n\;{\left(Z\;e\right)}^{2}$, and the Coulomb tunneling becomes easier.

In addition to this increment, we can introduce another one due to the negative entropy effect of plasma to the colliding pair. The expression of negentropy can be derived from internal and excess free energy given in [22,23]. Beyond the Debye-Hückel, other terms representing negative contributions to entropy permit the surrounding particles, while two nuclei are tunneling toward one another, to transfer energy making close approach easier against the repulsive Coulomb potential.

The same plasma negentropy effect can be introduced in measurements of correlations enhanced collision rates using cryogenic non-neutral plasmas [24].

- Fluctuations

As a final example that deserves further attention, let us now consider a part of the system itself instead of a subsystem of a different nature from the system or medium.

We divide the system of *N* elements in *W* cells of an elementary volume τ=h/π, where *h* is the Planck constant and π is the phase space momentum volume. The cell A may receive in time Δt the energy $Z\;T\;S^{-}$, being *Z* the number of elements interacting with the cell A (Z≪N). In a successive time Δt, the cell A can lose the energy received because other cells of the system enter into an interaction with it. Therefore, the energy of each element fluctuates based on the quantity $T\;S^{-}$. The time Δt is given by the uncertainty principle: $\Delta t\approx h/T\;S^{-}$. For instance, a system at kT=10eV, with $S^{-}=3.5\times 10^{-4}\;\mathrm{eV}/K$, we obtain $\Delta t=10^{-16}\;s$, that is the time between two elastic collisions of the cell. In this time, any element of the cell fluctuates in energy of the quantity $T\;S^{-}\approx 40\;\mathrm{eV}$. We conclude that the energy fluctuation of a system in a given time is induced by the presence of negentropy, which occurs because of the existence of correlations among the elements of the system, as it can be expected (all details on the evaluation of this effect will be given elsewhere).

A list of systems with important negative contributions to entropy is reported in works by Yi-Fang Chang [9,10]. Chang reported the decrease of entropy in macroscopic thermodynamics quantities in many different isolated systems submitted to gravitational interactions, attractive electromagnetic interactions, and strong nuclear interaction (weak nuclear being excluded). From microscopic nuclear, atomic, molecular, and nano-physical theories, one can obtain the decrease of entropy in macroscopic thermodynamics.

## 8. Conclusions

Since the early works by Szilard [25], Schrödinger [26] and Brillouin [27], many researchers have learned how useful it is to apply the concept of negentropy in many complex problems in physics, biology and information science. Later, Sato [7,8] has generalized the definition of negentropy and his last proposal was linked to the Kullback-Leibler approach to statistical mechanics [11,12]. This definition allows the use of negentropy not only in isolated systems but also in a wider class of systems. Yi-Fang Chang [9,10] has recently remarked that negentropy enters into the description of many different physical situations.

Our contribution in this paper is that by separating the positive from the negative contribution of entropy, we may suggest that a subsystem inserted into a non chaotic system can benefit from a certain amount of energy transferred by a group of elements of the system, those that within a certain amount of time enter into an interaction with the subsystem itself.

In a few cases we have made approximate calculations of the energy that each element of the system can transfer. The number of elements in the subsystem is much smaller than those of the system and interact with a small part of elements before participating in processes, like nuclear fusion and Coulomb tunnelling that, without the transferred energy, would be absent. The system in this transfer of energy through the negentropy does not change its bulk properties. We have evaluated in a few cases the amount of energy that can be transferred per element. Further applications to turbulent motion and screening problems of thermonuclear reactions are in progress.
